# Anacetrapib, a New CETP Inhibitor: The New Tool for the Management of Dyslipidemias?

**DOI:** 10.3390/diseases5040021

**Published:** 2017-09-29

**Authors:** Theodosios D. Filippatos, Anastazia Kei, Moses S. Elisaf

**Affiliations:** Department of Internal Medicine, School of Medicine, University of Ioannina, Ioannina 45110, Greece; keianastazia@gmail.com (A.K.); melisaf54@gmail.com (M.S.E.)

**Keywords:** anacetrapib, cholesteryl ester transfer protein, cardiovascular disease, apolipoprotein, diabetes

## Abstract

Cholesteryl ester transfer protein (CETP) inhibitors significantly increase serum high-density lipoprotein cholesterol (HDL) cholesterol levels and decrease low-density lipoprotein cholesterol (LDL) cholesterol concentration. However, three drugs of this class failed to show a decrease of cardiovascular events in high-risk patients. A new CETP inhibitor, anacetrapib, substantially increases HDL cholesterol and apolipoprotein (Apo) AI levels with a profound increase of large HDL2 particles, but also pre-β HDL particles, decreases LDL cholesterol levels mainly due to increased catabolism of LDL particles through LDL receptors, decreases lipoprotein a (Lp(a)) levels owing to a decreased Apo (a) production and, finally, decreases modestly triglyceride (TRG) levels due to increased lipolysis and increased receptor-mediated catabolism of TRG-rich particles. Interestingly, anacetrapib may be associated with a beneficial effect on carbohydrate homeostasis. Furthermore, the Randomized EValuation of the Effects of Anacetrapib Through Lipid-modification (REVEAL) trial showed that anacetrapib administration on top of statin treatment significantly reduces cardiovascular events in patients with atherosclerotic vascular disease without any significant increase of adverse events despite its long half-life. Thus, anacetrapib could be useful for the effective management of dyslipidemias in high-risk patients that do not attain their LDL cholesterol target or are statin intolerable, while its role in patients with increased Lp(a) levels remains to be established.

## 1. Introduction

Cholesteryl ester transfer protein (CETP) is a glycoprotein synthesized mainly in the liver, which plays a prominent role in the bidirectional transfer of cholesterol esters and triglycerides (TRG) between lipoproteins, that is the transfer of cholesterol esters from the cardioprotective high-density lipoprotein (HDL) particles to the potentially atherogenic non-HDL particles (very low-density lipoprotein (VLDL) particles, remnant lipoproteins and low-density lipoprotein (LDL) particles) [1,2]. Drugs that inhibit CETP are able to increase HDL cholesterol and also to decrease serum LDL cholesterol levels [3]. However, the randomized placebo-controlled trials that evaluated the effects of three drugs of this class, that is torcetrapib, dalcetrapib and evacetrapib, failed to show a beneficial cardiovascular effect [4,5,6,7]. These trials were prematurely terminated due to either off-target toxicity (torcetrapib) or lack of efficacy (dalcetrapib, evacetrapib) [4,5,6,7,8]. However, it has been recently reported that in the Randomized EValuation of the Effects of Anacetrapib Through Lipid-modification (REVEAL) trial, anacetrapib, a new CETP inhibitor, led to a significant decrease of cardiovascular events.

Thus, the aim of the review is to present the lipid/lipoprotein and cardiovascular effects of anacetrapib, as well as to discuss possible future indications of the drug.

## 2. Main Results of the REVEAL Trial

The REVEAL trial was a randomized placebo-controlled trial that assessed the efficacy and safety of anacetrapib in 30,449 adults with atherosclerotic vascular disease on intensive atorvastatin treatment (mean LDL cholesterol 61 mg/dL and mean non-HDL cholesterol 92 mg/dL) [9]. The administration of 100 mg of anacetrapib for a mean follow-up period of 4.1 years was associated with a significant reduction of the primary end point (first major coronary event) by 9% (rate ratio 0.91, 95% confidence interval 0.85–0.97, *p* = 0.004). This risk reduction was evident even though patients treated with anacetrapib exhibited slightly higher systolic blood pressure (SBP) and diastolic blood pressure (DBP) values (by 0.7/0.3 mm Hg, respectively) at the final visit compared with the control group. Treatment was well tolerated, and no significant differences between groups in the risk of death, cancer or other serious adverse events were observed. In fact, the incidence of new-onset diabetes mellitus among patients without diabetes mellitus at baseline was lower in the anacetrapib group as compared to the control group (5.3% vs. 6%, rate ratio 0.89, 95% CI 0.79–1.00, *p* = 0.0496) [9].

### 2.1. Effects of Anacetrapib on Lipid and Lipoprotein Profile

Anacetrapib is an inhibitor of CETP, which can lead to impressive changes of the serum lipid profile, as shown in Table 1, which includes the results of the REVEAL trial and two randomized trials that evaluated the efficacy of the drug in both high-risk patients, as well as in patients with familial hypercholesterolemia [9,10,11].

**Figure 1:**
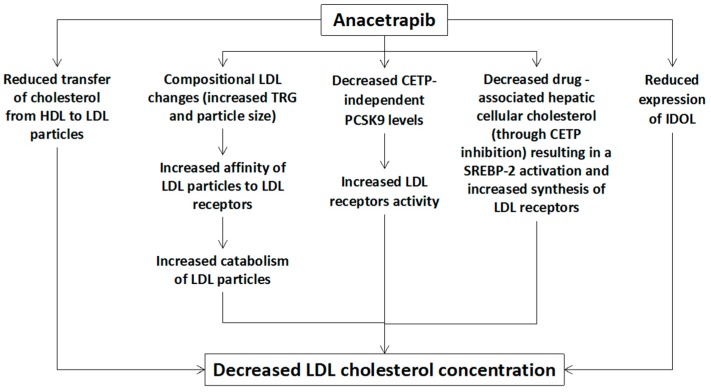
Potential mechanisms of anacetrapib-mediated reduction of low-density lipoprotein (LDL) cholesterol. HDL: high-density lipoprotein, LDL: low-density lipoprotein, CETP: cholesteryl ester transfer protein, TRG: triglycerides, SREBP: sterol regulatory element binding protein, IDOL: inducible degrader of the low-density lipoprotein receptor, PCSK9: proprotein convertase subtilisin/kexin type 9.

Beyond the prominent increase of HDL cholesterol, a marked decrease of LDL cholesterol was observed in these trials. Even though the underlying mechanisms of this increase in LDL cholesterol are not clear, kinetic data of lipid metabolism during anacetrapib administration in humans points to the following potential mechanisms (Figure 1) [12,13,14,15]:

(1) Increased catabolism of LDL particles due to anacetrapib-associated compositional changes of LDL particles, such as increased TRG content and particle size, leading to increased LDL particles’ affinity to the LDL receptors [15].

(2) Decreased proprotein convertase subtilisin/kexin type 9 (PCSK9) levels (a CETP-independent mechanism) leading to decreased LDL receptors’ degradation and, therefore, increased LDL receptors’ activity and LDL particles’ catabolism [14,16].

(3) Decreased drug-associated cellular cholesterol concentration in the liver, due to increased cholesterol efflux, which results in the activation of sterol regulatory element-binding protein 2 (SREBP-2) leading to increased synthesis of LDL receptors [15].

(4) Reduced expression of the inducible degrader of the LDL receptors (IDOL) due to decreased hepatic oxysterols resulting in a reduced activation of liver X receptors (LRX). This reduced IDOL expression is associated with increased LDL receptors’ activity and subsequently with increased catabolism of LDL particles [15].

(5) Reduced transfer of cholesterol esters from HDL to LDL is also associated with a reduction of LDL cholesterol, though this mechanism cannot explain the observed marked reduction of apolipoprotein (Apo) B levels that reflects a reduction in the concentration of LDL particles [12,15].

Thus, many mechanisms may explain the LDL cholesterol-lowering efficacy of anacetrapib. LDL cholesterol is an established cardiovascular risk factor and represents the main target of hypolipidemic therapy [17,18]. Furthermore, many of the above mechanisms explain the anacetrapib-associated lowering of Apo B levels (Table 1). In this context, the findings of the REVEAL trial were in accordance with the results of the Cholesterol Treatment Trialists Collaboration meta-analysis (*n* = 170,000 participants) [19], which showed that a reduction of non-HDL cholesterol (which represents the Apo B-containing particles) by 17 mg/dL is expected to reduce the rate of major coronary events by 10%. In fact, recent Mendelian randomization studies have shown that Apo B is a better than LDL cholesterol predictor of an increased cardiovascular risk in patients with gene variants that reflect combined CETP inhibitor and statin treatment [20]. Therefore, it has been proposed that the anacetrapib-mediated decrease of Apo B is the main mechanism of cardiovascular risk reduction in patients taking a statin [20].

A decrease of lipoprotein a (Lp(a)) levels was repeatedly observed after anacetrapib and other CETP inhibitors’ administration (Table 1) [9,10,11]. Interestingly, a recently published study clearly showed that the anacetrapib-mediated reduction of Lp(a) levels (by 34.1%) is due to a reduction of the Apo (a) production rate (by 41%) and not due to changes of Apo (a) fractional catabolic rate [21]. Lp(a) is an established cardiovascular risk factor [22]; thus, the anacetrapib-mediated decrease of its serum concentration may have played a significant role in the positive results of the REVEAL trial.

**Figure 2:**
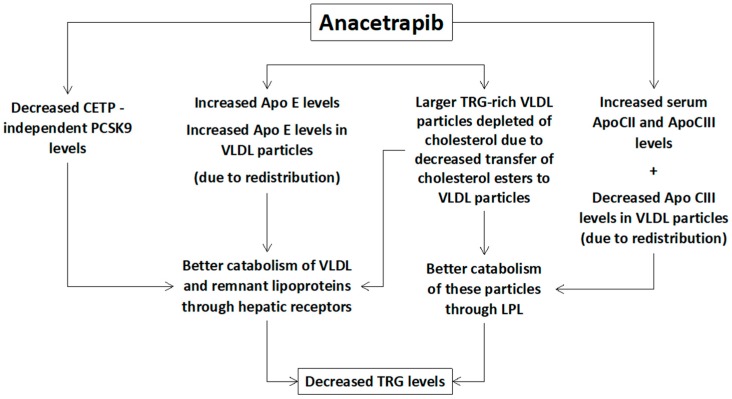
Potential mechanisms of the anacetrapib-mediated reduction of triglycerides (TRG). PCSK9: proprotein convertase subtilisin/kexin type 9, CETP: cholesteryl ester transfer protein, Apo: apolipoprotein, LPL: lipoprotein lipase, VLDL: very low-density lipoprotein.

Additionally, a small decrease of serum TRG levels is also observed with anacetrapib [9,10,11] mainly due to increased catabolism of TRG-rich VLDL particles (Figure 2). The underlying mechanisms include:

(1) Increased lipolysis of the large TRG-rich VLDL particles through lipoprotein lipase (even without increased lipolytic activity) [23].

(2) Other compositional changes in VLDL particles, such as increased Apo E and reduced Apo CIII content, which can increase lipoprotein lipase activity and the hepatic receptor-mediated clearance of remnant particles [12,23].

(3) Increased hepatic uptake of the large triglyceride-rich (depleted of cholesterol) VLDL particles [16,23].

(4) CETP-independent decreased PCSK9 levels leading to increased LDL receptors’ activity and catabolism of VLDL particles and their remnants [16].

**Figure 3:**
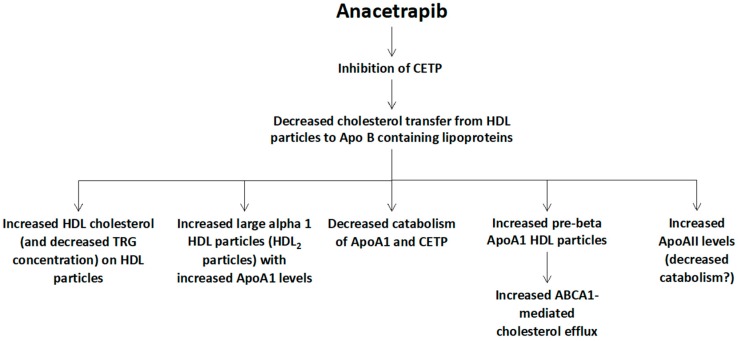
Potential mechanisms of anacetrapib-mediated changes of high-density lipoprotein (HDL) metabolism. CETP: cholesteryl ester transfer protein, Apo: apolipoprotein, TRG: triglycerides, ABCA1: ATP-binding cassette transporter ABCA1 (member 1 of human transporter sub-family ABCA).

Finally, a number of trials has delineated the anacetrapib-mediated changes of HDL particles, which include a marked increase of HDL cholesterol, an increase of Apo AI levels (due to its decreased catabolism) and a lesser increase of Apo AII levels, as well as an increase of large cholesterol-rich alpha 2 HDL particles (HDL2 particles) and pre-beta HDL particles, which can lead to an increased ATP-binding cassette transporter (ABCA1)-mediated cholesterol efflux (Figure 3) [24].

### 2.2. Other Effects of Anacetrapib

In contrast with the other drugs of this class, anacetrapib does not seem to exhibit significant ‘’on-target’’ adverse effects, such as dysfunctional HDL particles or changes in apolipoproteins that promote atherogenesis, or ‘’off target’’ effects, such as a significant increase of blood pressure or C-reactive protein levels (which is an inflammatory marker) [8,25]; these adverse effects have been implicated in the negative or neutral cardiovascular effects of other CETP inhibitors. However, in the REVEAL trial, slightly higher SBP and DBP values (by 0.7/0.3 mm Hg, respectively) were observed at the final visit in anacetrapib-treated patients compared with the control group. Alternatively, any on-target or off-target detrimental effects of anacetrapib may have been counterbalanced by the marked decrease of Apo B-containing atherogenic lipoproteins, such as LDL, but also Lp(a) [9].

Mendelian randomization trials have shown that hypolipidemic drugs that reduce LDL receptors’ activity are associated with a detrimental effect on carbohydrate homeostasis [26,27,28,29], which has received much attention taking into account the wide use of these hypolipidemic drugs. The beneficial effect of CETP inhibitors on glucose homeostasis, as shown in the REVEAL trial and previous studies [9,30], can possibly counteract the dysglycemic effect of other hypolipidemic drugs. Thus, anacetrapib could be a promising therapeutic strategy as an add-on therapy to current hypolipidemic treatment especially in high-risk patients with disorders of carbohydrate metabolism.

It should be mentioned that anacetrapib has a long half-life, which is associated with advantages concerning the patients’ compliance to therapy, but may be a disadvantage concerning side effects that can be observed after long-term treatment [31].

## 3. Possible Future Indications of Anacetrapib

Based on the current knowledge regarding its effects on lipidemic profile and cardiovascular risk, anacetrapib could be useful for the treatment of:

(1) High-risk patients who do not attain their LDL cholesterol target despite optimal hypolipidemic treatment. Currently, these patients could take a PCSK9 inhibitor [32,33], but anacetrapib could be alternatively used taking into account that it is associated with a reduction of cardiovascular events, is given orally once per day (in contrast with the parenteral administration of current PCSK9 inhibitors) and probably will have a much lower cost.

(2) Patients with familial hypercholesterolemia with difficulty achieving their LDL cholesterol target despite optimal hypolipidemic treatment, in whom anacetrapib is associated with a significant improvement of lipid and lipoprotein profile (Table 1).

(3) Patients with statin intolerance, in whom the use of the expensive and parenteral PCSK9 inhibitors is not easily accepted. This possible indication of anacetrapib could be the most important in the future since non-statin treatment with a potent CETP inhibitor (possibly in combination with ezetimibe) could be associated with large concordant absolute reductions of both LDL cholesterol and Apo B and, therefore, of cardiovascular risk [20].

(4) Patients with increased serum Lp(a) levels, although more data are needed in this population.

It is worth mentioning that two new CETP inhibitors that induce significant beneficial changes in lipidemic profile (including a reduction of Apo B-containing lipoproteins) are under investigation [Obicetrapib (AMG-899, TA-8995) and K-312] [34,35]. The results of the relative studies will show whether there is a class effect of CETP inhibitors on cardiovascular events and will help to delineate the implicated mechanisms.

## 4. Conclusions

Anacetrapib is associated with significant improvement of lipid and lipoprotein profile and a significant reduction of cardiovascular events in high-risk patients on optimal statin treatment. These data may suggest that anacetrapib could be a useful therapeutic option for the effective hypolipidemic treatment of high-risk patients especially if the results of ongoing trials with other CETP inhibitors confirm the positive cardiovascular effects of this drug class.

## Figures and Tables

**Table 1:** Anacetrapib (100 mg/d)-associated percentage changes of lipid parameters versus placebo in randomized clinical trials.

Lipid Parameters	REALIZE Trial [11] (Patients with Familial Hypercholesterolemia)	DEFINE Trial [10] (Patients with Coronary Heart Disease or at Risk for Coronary Heart Disease)	REVEAL Trial [9] (Patients with Atherosclerotic Vascular Disease) *
LDL cholesterol	−39.7% **	−39.8% ***	−41% (direct method) −17% ** (in a subgroup of 2000 patients)
HDL cholesterol	+102.1%	+138.1%	+104%
Non-HDL cholesterol	−36.4%	−31.7%	−18%
Apo B	−24.8%	−21%	−18%
Apo AI	+32.9%	+44.7%	+36%
Lp(a)	−27.9%	−36.4%	−25%
TRG	−5.5%	−6.8%	−7%

* Lipid levels at the trial midpoint. ** Measured by β quantification. *** LDL-cholesterol levels were calculated with the use of the Friedewald equation: LDL cholesterol = total cholesterol − (HDL cholesterol + [triglycerides ÷ 5]). If the triglyceride level was more than 400 mg per deciliter, LDL-cholesterol was measured by means of preparative ultracentrifugation separation. REALIZE: Randomized Evaluation of Anacetrapib Lipid-Modifying Therapy in Patients with Heterozygous Familial Hypercholesterolemia, DEFINE: Determining the EFficacy and Tolerability of CETP INhibition with AnacEtrapib, REVEAL: Randomized EValuation of the Effects of Anacetrapib Through Lipid-modification, LDL: low-density lipoprotein, HDL: high-density lipoprotein, Apo: apolipoprotein, Lp(a): lipoprotein (a), TRG: triglycerides.

## References

[1] Barter P.J., Rye K.A. (2012). Cholesteryl ester transfer protein inhibition as a strategy to reduce cardiovascular risk. J. Lipid. Res..

[2] Tall A.R. (1993). Plasma cholesteryl ester transfer protein. J. Lipid. Res..

[3] Barter P.J., Rye K.A. (2016). Cholesteryl Ester Transfer Protein Inhibition Is Not Yet Dead-Pro. Arterioscler Thromb Vasc. Biol..

[4] Barter P.J., Caulfield M., Eriksson M., Grundy S.M., Kastelein J.J., Komajda M., Lopez-Sendon J., Mosca L., Tardif J.C., Waters D.D. (2007). Effects of torcetrapib in patients at high risk for coronary events. N. Engl. J. Med..

[5] Schwartz G.G., Olsson A.G., Abt M., Ballantyne C.M., Barter P.J., Brumm J., Chaitman B.R., Holme I.M., Kallend D., Leiter L.A. (2012). Effects of dalcetrapib in patients with a recent acute coronary syndrome. N. Engl. J. Med..

[6] Lincoff A.M., Nicholls S.J., Riesmeyer J.S., Barter P.J., Brewer H.B., Fox K.A.A., Gibson C.M., Granger C., Menon V., Montalescot G. (2017). Evacetrapib and Cardiovascular Outcomes in High-Risk Vascular Disease. N. Engl. J. Med..

[7] Filippatos T.D., Elisaf M.S. (2017). Evacetrapib and cardiovascular outcomes: Reasons for lack of efficacy. J. Thoracic Disease.

[8] Filippatos T.D., Klouras E., Barkas F., Elisaf M. (2016). Cholesteryl ester transfer protein inhibitors: Challenges and perspectives. Expert. Rev. Cardiovasc. Ther..

[9] HPS3/TIMI55-REVEAL Collaborative Group (2017). Effects of Anacetrapib in Patients with Atherosclerotic Vascular Disease. N. Engl. J. Med..

[10] Cannon C.P., Shah S., Dansky H.M., Davidson M., Brinton E.A., Gotto A.M., Stepanavage M., Liu S.X., Gibbons P., Ashraf T.B. (2010). Safety of anacetrapib in patients with or at high risk for coronary heart disease. N. Engl. J. Med..

[11] Kastelein J.J., Besseling J., Shah S., Bergeron J., Langslet G., Hovingh G.K., Al-Saady N., Koeijvoets M., Hunter J., Johnson-Levonas A.O. (2015). Anacetrapib as lipid-modifying therapy in patients with heterozygous familial hypercholesterolaemia (REALIZE): A randomised, double-blind, placebo-controlled, phase 3 study. Lancet.

[12] Millar J.S., Lassman M.E., Thomas T., Ramakrishnan R., Jumes P., Dunbar R.L., de Goma E.M., Baer A.L., Karmally W., Donovan D.S. (2017). Effects of CETP inhibition with anacetrapib on metabolism of VLDL-TG and plasma apolipoproteins C-II, C-III, and E. J. Lipid Res..

[13] Krauss R.M., Pinto C.A., Liu Y., Johnson-Levonas A.O., Dansky H.M. (2015). Changes in LDL particle concentrations after treatment with the cholesteryl ester transfer protein inhibitor anacetrapib alone or in combination with atorvastatin. J. Clin. Lipidol..

[14] Barter P.J., Tabet F., Rye K.A. (2015). Reduction in PCSK9 levels induced by anacetrapib: An off-target effect?. J. Lipid Res..

[15] Millar J.S., Reyes-Soffer G., Jumes P., Dunbar R.L., deGoma E.M., Baer A.L., Karmally W., Donovan D.S., Rafeek H., Pollan L. (2015). Anacetrapib lowers LDL by increasing ApoB clearance in mildly hypercholesterolemic subjects. J. Clin. Investig..

[16] Van der Tuin S.J., Kuhnast S., Berbee J.F., Verschuren L., Pieterman E.J., Havekes L.M., van der Hoorn J.W., Rensen P.C., Jukema J.W., Princen H.M. (2015). Anacetrapib reduces (V)LDL cholesterol by inhibition of CETP activity and reduction of plasma PCSK9. J. Lipid Res..

[17] Filippatos T.D., Elisaf M.S. (2015). Are lower levels of LDL-cholesterol really better? Looking at the results of IMPROVE-IT: Opinions of three experts—III. Hellenic. J. Cardiol..

[18] Catapano A.L., Graham I., De Backer G., Wiklund O., Chapman M.J., Drexel H., Hoes A.W., Jennings C.S., Landmesser U., Pedersen T.R. (2016). 2016 ESC/EAS Guidelines for the Management of Dyslipidaemias. Eur. Heart J..

[19] Baigent C., Blackwell L., Emberson J., Holland L.E., Reith C., Bhala N., Peto R., Barnes E.H., Keech A., Simes J. (2010). Efficacy and safety of more intensive lowering of LDL cholesterol: A meta-analysis of data from 170,000 participants in 26 randomised trials. Lancet.

[20] Ference B.A., Kastelein J.J.P., Ginsberg H.N., Chapman M.J., Nicholls S.J., Ray K.K., Packard C.J., Laufs U., Brook R.D., Oliver-Williams C. (2017). Association of Genetic Variants Related to CETP Inhibitors and Statins With Lipoprotein Levels and Cardiovascular Risk. JAMA.

[21] Thomas T., Zhou H., Karmally W., Ramakrishnan R., Holleran S., Liu Y., Jumes P., Wagner J.A., Hubbard B., Previs S.F. (2017). CETP (Cholesteryl Ester Transfer Protein) Inhibition With Anacetrapib Decreases Production of Lipoprotein(a) in Mildly Hypercholesterolemic Subjects. Arterioscler Thromb. Vasc. Biol..

[22] Milionis H.J., Filippatos T.D., Loukas T., Bairaktari E.T., Tselepis A.D., Elisaf M.S. (2006). Serum lipoprotein(a) levels and apolipoprotein(a) isoform size and risk for first-ever acute ischaemic nonembolic stroke in elderly individuals. Atherosclerosis.

[23] Brown A.L., Brown J.M. (2017). Anacetrapib-driven triglyceride lowering explained: The fortuitous role of CETP in the intravascular catabolism of triglyceride-rich lipoproteins. J. Lipid Res..

[24] Reyes-Soffer G., Millar J.S., Ngai C., Jumes P., Coromilas E., Asztalos B., Johnson-Levonas A.O., Wagner J.A., Donovan D.S., Karmally W. (2016). Cholesteryl Ester Transfer Protein Inhibition With Anacetrapib Decreases Fractional Clearance Rates of High-Density Lipoprotein Apolipoprotein A-I and Plasma Cholesteryl Ester Transfer Protein. Arterioscler Thromb. Vasc. Biol..

[25] Raal F.J., Blom D.J. (2015). Anacetrapib in familial hypercholesterolaemia: Pros and cons. Lancet.

[26] Filippatos T.D., Filippas-Ntekouan S., Pappa E., Panagiotopoulou T., Tsimihodimos V., Elisaf M.S. (2017). PCSK9 and carbohydrate metabolism: A double-edged sword. World J. Diabetes.

[27] Lotta L.A., Sharp S.J., Burgess S., Perry J.R.B., Stewart I.D., Willems S.M., Luan J., Ardanaz E., Arriola L., Balkau B. (2016). Association Between Low-Density Lipoprotein Cholesterol-Lowering Genetic Variants and Risk of Type 2 Diabetes: A Meta-analysis. JAMA.

[28] Filippatos T.D., Elisaf M.S. (2016). Effects of ezetimibe/simvastatin combination on metabolic parameters. Int. J. Cardiol..

[29] Filippatos T.D., Elisaf M.S. (2016). Pitavastatin and carbohydrate metabolism: What is the evidence?. Expert Rev. Clin. Pharmacol.

[30] Barter P.J., Rye K.A., Tardif J.C., Waters D.D., Boekholdt S.M., Breazna A., Kastelein J.J. (2011). Effect of torcetrapib on glucose, insulin, and hemoglobin A1c in subjects in the Investigation of Lipid Level Management to Understand its Impact in Atherosclerotic Events (ILLUMINATE) trial. Circulation.

[31] Borghi C., Cicero A.F. (2017). Pharmacokinetic drug evaluation of anacetrapib for the treatment of dyslipidemia. Expert Opin. Drug Metab. Toxicol..

[32] Sabatine M.S., Giugliano R.P., Keech A.C., Honarpour N., Wiviott S.D., Murphy S.A., Kuder J.F., Wang H., Liu T., Wasserman S.M. (2017). Evolocumab and Clinical Outcomes in Patients with Cardiovascular Disease. N. Engl. J. Med..

[33] Filippatos T.D., Kei A., Rizos C.V., Elisaf M.S. (2017). Effects of PCSK9 Inhibitors on Other than Low-Density Lipoprotein Cholesterol Lipid Variables. J. Cardiovasc. Pharmacol Ther..

[34] Hovingh G.K., Kastelein J.J., Van Deventer S.J., Round P., Ford J., Saleheen D., Rader D.J., Brewer H.B., Barter P.J. (2015). Cholesterol ester transfer protein inhibition by TA-8995 in patients with mild dyslipidaemia (TULIP): A randomised, double-blind, placebo-controlled phase 2 trial. Lancet.

[35] Miyosawa K., Watanabe Y., Murakami K., Murakami T., Shibata H., Iwashita M., Yamazaki H., Yamazaki K., Ohgiya T., Shibuya K. (2015). New CETP inhibitor K-312 reduces PCSK9 expression: A potential effect on LDL cholesterol metabolism. Am. J. Physiol. Endocrinol. Metab..

